# Pulsatility as a Potential Regulator of Cardiovascular Biology: Molecular, Cellular, and Hemodynamic Remodeling During Continuous-Flow Left Ventricular Assist Device Support and Following Heart Transplantation

**DOI:** 10.3390/ijms27156650

**Published:** 2026-07-25

**Authors:** Przemysław Lutomski, Calogera Pisano, Krzysztof J. Filipiak, Giuseppe Maria Raffa, Roberta Vazzana, Ewelina Grywalska, Mansur Rahnama, Mariusz Kowalewski, Małgorzata Tomaszewska, Piotr Suwalski, Zbigniew Krasiński, Marek Jemielity, Jacek Zieliński, Tomasz Urbanowicz

**Affiliations:** 1 Chair of Physiotherapy, Department of Sport Medicine and Traumatology, Faculty of Health Sciences, Poznan University of Physical Education, 27/39 Królowej Jadwigi Street, 61-871 Poznan, Poland; 2 Department of Research, IRCCS-ISMETT (Mediterranean Institute for Transplantation and Specialized Therapies), 90127 Palermo, Italy; 3 Department of Precision Medicine in Medical Surgical and Critical Area (Me.Pre.C.C.), University of Palermo, 90134 Palermo, Italy; 4 The Center of Postgraduate Medical Education, 99/103 Marymoncka Street, 01-813 Warsaw, Poland; 5 Department of Experimental Immunology, Faculty of Medical Sciences, Medical University of Lublin, 6 Chodźki Street, 20-093 Lublin, Poland; 6 Department of Dental Surgery, Medical University of Lublin, 6 Chodźki Street, 20-093 Lublin, Poland; 7 Department of Cardiac Surgery, Center of Postgraduate Medical Education, Central Clinical Hospital of the Ministry of Interior, 02-507 Warszawa, Poland; 8 Central Clinical Hospital, Poznan University of Medical Sciences, 49 Przybyszewskiego Street, 60-355 Poznan, Poland; 9 Department of Vascular, Endovascular Surgery, Angiology and Phlebology, Poznan University of Medical Sciences, 1/2 Dluga Street, 61-848 Poznan, Poland; 10 Cardiac Surgery and Transplantology Department, Poznan University of Medical Sciences, 1/2 Długa, 61-848 Poznan, Poland; 11 Department of Athletics, Strength and Conditioning, Poznan University of Physical Education, 27/39 Królowej Jadwigi Street, 61-871 Poznan, Poland

**Keywords:** heart transplantation, LVAD, pulsatile flow

## Abstract

Pulsatile blood flow is a fundamental characteristic of cardiovascular physiology that regulates endothelial function, vascular homeostasis, microcirculatory integrity, and organ adaptation through complex mechanobiological pathways. The widespread use of continuous-flow left ventricular assist devices (CF-LVADs) has created a unique clinical model of chronic pulsatility deprivation, whereas heart transplantation restores physiological pulsatile hemodynamics. This review examines the molecular, cellular, and systemic consequences of these contrasting circulatory states. Evidence from experimental and clinical studies indicates that reduced pulsatility during CF-LVAD support is associated with impaired endothelial mechanotransduction, glycocalyx disruption, oxidative stress, inflammatory activation, angiogenic dysregulation, acquired von Willebrand syndrome, and microvascular remodeling. These alterations contribute to bleeding, thrombosis, neurological events, and progressive end-organ dysfunction. In contrast, restoration of pulsatile flow following heart transplantation promotes recovery of endothelial signaling, nitric oxide bioavailability, vascular responsiveness, and tissue perfusion, although persistent immune-mediated injury may limit complete vascular normalization. Emerging concepts involving Piezo1 signaling, YAP/TAZ mechanotransduction, extracellular vesicles, immunometabolism, and multi-omics profiling further support the role of pulsatility as a biological regulator rather than a simple hemodynamic consequence of cardiac contraction. Understanding pulsatility-dependent cardiovascular remodeling may facilitate the development of next-generation circulatory support technologies and novel therapeutic strategies to preserve vascular health.

## 1. Introduction

The evolution of mechanical circulatory support has transformed the treatment landscape of advanced heart failure [1]. Cardiovascular diseases remain the leading cause of death worldwide, accounting for approximately 20 million deaths annually and affecting more than 500 million individuals [2]. Among these conditions, heart failure affects over 64 million people globally and represents one of the fastest-growing causes of hospitalization, disability, and healthcare expenditure [3,4]. Despite substantial advances in pharmacological and device-based therapies, advanced heart failure continues to carry an extremely poor prognosis, making durable mechanical circulatory support and heart transplantation indispensable therapeutic options for carefully selected patients [5,6].

Continuous-flow left ventricular assist devices (CF-LVADs) have become the predominant form of durable mechanical support, offering prolonged survival and improved quality of life for patients with end-stage cardiac disease [7,8]. Simultaneously, heart transplantation remains the definitive treatment for eligible patients, restoring physiological cardiac architecture and normal circulatory dynamics [9]. While both therapeutic strategies effectively improve systemic perfusion and reverse many manifestations of advanced heart failure, they establish fundamentally different hemodynamic environments.

The human cardiovascular system evolved under conditions of pulsatile flow generated by cyclic ventricular contraction [10]. Pulsatility is not merely a mechanical consequence of cardiac function but rather a biological signal that regulates endothelial phenotype, vascular tone, coagulation, inflammatory responses, microvascular perfusion, and organ homeostasis [11]. The widespread adoption of CF-LVAD technology has created an unprecedented physiological experiment in which humans survive for years under conditions of markedly reduced pulsatility [12]. In contrast, heart transplantation restores physiological pulse pressure and re-establishes the biomechanical signals that have shaped cardiovascular evolution.

Most previous studies have examined LVAD-related complications or post-transplant outcomes independently [13,14,15,16]. However, few investigations have considered these treatment modalities as opposite ends of a unique biological continuum defined by the presence or absence of pulsatile hemodynamics. Such a perspective may provide novel insights into the molecular mechanisms governing vascular adaptation and maladaptation.

The central hypothesis of this review is that pulsatility may function as an important regulator of cardiovascular biology, influencing vascular adaptation through interconnected mechanotransduction, inflammatory, metabolic, and hemodynamic pathways. Continuous-flow support induces a distinct molecular phenotype characterized by altered mechanotransduction, endothelial dysfunction, inflammatory activation, coagulation abnormalities, and microvascular remodeling, whereas restoration of pulsatile circulation following transplantation partially reverses these changes. Understanding these mechanisms may facilitate the development of future circulatory support technologies that preserve the biological advantages of physiological pulsatility while maintaining the durability of contemporary mechanical pumps.

An underappreciated concept is that pulsatility may be a more important contributor than a mere consequence of ventricular contraction. Throughout vertebrate evolution, vascular cells adapted to rhythmic fluctuations in pressure, stretch, and shear stress generated by the beating heart [17]. Consequently, endothelial and smooth muscle cells developed mechanosensory systems specifically tuned to cyclic hemodynamic stimuli [18].

The widespread clinical implementation of continuous-flow LVADs has therefore created a unique physiological state in which blood flow is restored while one of the most fundamental biological signals in cardiovascular evolution is largely removed [19]. Conversely, heart transplantation restores not only cardiac output but also the pulsatile biomechanical environment under which vascular homeostasis evolved. From this perspective, LVAD support and heart transplantation can be viewed as complementary human models for studying the biological consequences of pulsatility deprivation and restoration. This paradigm forms the conceptual foundation of the present review.

Although continuous-flow LVAD support and heart transplantation are both established therapies for advanced heart failure, they create fundamentally distinct hemodynamic and biological environments [20]. Most previous investigations have examined these treatment modalities separately, focusing either on device-related complications or post-transplant outcomes [21]. However, an emerging body of evidence suggests that many of the observed differences may be explained by a common underlying determinant: the presence or absence of physiological pulsatility. The concept that pulsatile flow regulates vascular, cellular, and molecular homeostasis provides a unifying framework for interpreting seemingly disparate clinical phenomena—including endothelial dysfunction, bleeding complications, angiogenic activation, inflammatory remodeling, and organ-specific adaptation [22]. To establish this conceptual foundation, the principal biological consequences of chronic pulsatility deprivation during continuous-flow LVAD support and pulsatility restoration following heart transplantation are summarized in Table 1.

Although the biological domains presented in Table 1 are discussed separately throughout this review for clarity, they function as highly interconnected components of an integrated mechanobiological network. Endothelial dysfunction amplifies oxidative stress, which further enhances inflammatory activation and mitochondrial injury [51,52]. These processes promote angiogenic dysregulation, impair microvascular function, and influence neurohormonal signaling, ultimately contributing to progressive organ remodeling. Conversely, restoration of physiological pulsatility after heart transplantation improves multiple interconnected pathways rather than reversing each biological process in isolation. This coordinated systems-level response suggests that the biological effects of pulsatility arise from synergistic interactions among endothelial, inflammatory, metabolic, hemostatic, and microvascular mechanisms rather than isolated molecular events.

### 1.1. Literature Search Strategy

This narrative review was developed through a structured literature search performed in PubMed, Scopus, and Web of Science through June 2025. Search terms included “continuous-flow LVAD”, “heart transplantation”, “pulsatility”, “mechanotransduction”, “endothelial dysfunction”, “glycocalyx”, “vascular biology”, “microcirculation”, and “vascular remodeling”. Relevant original studies, translational investigations, and review articles published in English were evaluated. Particular emphasis was placed on studies examining molecular mechanisms linking altered hemodynamics to vascular adaptation.

### 1.2. Hemodynamic Remodeling During Continuous-Flow Circulation

Although the biological consequences of reduced pulsatility are the primary focus of this review, these alterations stem from profound changes in blood-flow dynamics induced by continuous-flow LVAD support. Under physiological conditions, ventricular ejection generates pulsatile pressure waves accompanied by highly organized helical and vortical flow structures throughout the left ventricle, aortic root, and large arteries [53,54]. These complex flow patterns improve energy efficiency, facilitate coronary perfusion, minimize flow stagnation, and generate spatially and temporally regulated wall shear stress that preserves endothelial homeostasis. Helical flow also contributes to the stabilization of laminar blood transport, reduces flow separation at arterial bifurcations, and promotes homogeneous distribution of mechanical forces across the vascular wall [55,56]. Collectively, these biomechanical characteristics represent essential determinants of normal cardiovascular physiology rather than simple hydrodynamic phenomena.

Continuous-flow LVADs fundamentally modify this physiological environment by generating nearly continuous forward flow with markedly reduced pulse pressure and attenuated oscillatory shear stress [23]. Although these devices effectively restore systemic perfusion, they alter vortex formation within the left ventricle and ascending aorta, reduce physiological helical flow, and expose blood elements to sustained high shear forces within the pump itself [57]. Importantly, the high shear stress responsible for von Willebrand factor degradation differs fundamentally from the low-pulsatility arterial environment that promotes endothelial dysfunction, impairs mechanotransduction, and leads to abnormal vascular adaptation [58]. Thus, biological remodeling during continuous-flow support reflects the combined effects of altered global hemodynamics, disrupted physiological flow architecture, and molecular responses to chronic pulsatility deprivation, providing the mechanistic foundation for the pathways discussed throughout the following sections.

Unlike previous reviews focusing primarily on device-related complications or transplant outcomes, the present review integrates mechanobiology, endothelial biology, immunometabolism, organ-specific adaptation, and systems-level molecular profiling into a unified conceptual framework in which pulsatility is considered a central regulator of cardiovascular biology rather than merely a hemodynamic characteristic [24,59,60].

Although pulsatility represents the principal mechanobiological concept developed throughout this review, cardiovascular remodeling during continuous-flow LVAD support results from interactions among multiple biological and device-related factors. Distinguishing mechanisms primarily attributable to reduced pulsatility from those related to advanced heart failure, pump-specific characteristics, and patient heterogeneity is essential for interpreting experimental findings and designing future mechanistic studies. The principal determinants contributing to cardiovascular remodeling during long-term mechanical circulatory support are summarized in Table 2.

## 2. Molecular Consequences of Pulsatility Loss During Continuous-Flow LVAD Support

### 2.1. Endothelial Mechanotransduction in the Absence of Physiological Pulsatility

Endothelial cells continuously translate hemodynamic forces into biochemical signals through a highly coordinated mechanosensory apparatus composed of the glycocalyx, integrins, PECAM-1, VE-cadherin complexes, primary cilia, and mechanosensitive ion channels. Under physiological conditions, pulsatile shear stress promotes an anti-inflammatory and antithrombotic endothelial phenotype characterized by activation of endothelial nitric oxide synthase (eNOS), Krüppel-like factor 2 (KLF2), and Krüppel-like factor 4 (KLF4) [78,79].

The transition to continuous-flow circulation fundamentally alters these signaling pathways. Reduced oscillatory pressure gradients and diminished pulse pressure decrease cyclic endothelial stimulation, leading to impaired nitric oxide production and diminished activation of protective transcriptional programs [80,81]. In physiological arteries, endothelial cells continuously decode cyclic fluctuations in shear stress, circumferential stretch, and pulse pressure to maintain vascular homeostasis [82,83]. Continuous-flow circulation attenuates these oscillatory mechanical signals, thereby reducing activation of mechanosensitive structures, including the glycocalyx, PECAM-1/VE-cadherin complexes, integrins, and Piezo1 ion channels [84]. Consequently, downstream activation of KLF2, KLF4, and endothelial nitric oxide synthase becomes attenuated, whereas pro-inflammatory pathways involving NF-κB, oxidative stress, and endothelial activation become increasingly dominant [85,86]. Thus, continuous flow not only removes pulsatility but also actively reprograms endothelial mechanotransduction toward a maladaptive biological phenotype.

Simultaneously, chronic exposure to non-physiological shear forces activates pro-inflammatory signaling cascades involving nuclear factor-κB (NF-κB), mitogen-activated protein kinases, and reactive oxygen species [87].

Mechanosensitive molecules such as Piezo1 and YAP/TAZ may represent central mediators of vascular adaptation during CF-LVAD support. Altered activation of these pathways influences endothelial survival, vascular permeability, and cellular metabolism, ultimately contributing to a unique vascular phenotype not observed in either healthy individuals or patients with untreated heart failure.

Recent studies have identified the Piezo1–YAP/TAZ signaling axis as a central regulator of endothelial adaptation to mechanical forces [88]. Piezo1 channels convert shear stress into intracellular calcium signals that subsequently modulate transcriptional programs controlling vascular homeostasis. Downstream activation of YAP and TAZ integrates biomechanical information with cellular metabolism, proliferation, and inflammatory responses. Under physiological pulsatile conditions, coordinated activation of Piezo1, YAP/TAZ, KLF2, and eNOS promotes an anti-inflammatory endothelial phenotype [89]. Loss of physiological pulsatility may therefore result in maladaptive mechanotransduction characterized by aberrant calcium signaling, impaired nitric oxide production, and transcriptional reprogramming toward vascular dysfunction. The Piezo1–YAP/TAZ pathway may represent one of the most important molecular links connecting altered hemodynamics to clinical complications observed during long-term mechanical circulatory support [90].

Despite compelling experimental evidence, direct investigations of Piezo1 signaling in patients supported with continuous-flow LVADs remain limited. Consequently, the contribution of Piezo1-mediated mechanotransduction to clinical complications remains largely inferential and requires further validation.

#### Endothelial Glycocalyx as the Primary Sensor of Pulsatile Flow

The endothelial glycocalyx is increasingly recognized as the first anatomical structure to be altered by pulsatile hemodynamics [91,92]. This dynamic carbohydrate-rich layer, composed primarily of syndecans, glypicans, heparan sulfate proteoglycans, and hyaluronic acid, functions as a biomechanical interface between circulating blood and endothelial cells. Physiological pulsatile flow induces cyclical deformation of the glycocalyx, thereby activating intracellular signaling pathways that regulate nitric oxide synthesis, vascular permeability, leukocyte adhesion, and endothelial survival. Experimental evidence suggests that disruption of glycocalyx integrity impairs mechanotransduction and promotes a pro-inflammatory vascular phenotype.

Chronic exposure to continuous-flow circulation may alter glycocalyx architecture through persistent non-physiological shear stress and oxidative injury [93]. The shedding of glycocalyx components, such as syndecan-1, has been associated with endothelial dysfunction, impaired microvascular regulation, and adverse cardiovascular outcomes. Whether restoration of physiological pulsatility following heart transplantation facilitates glycocalyx regeneration remains largely unexplored and represents a promising area for future investigation. Understanding glycocalyx biology may provide important mechanistic insights linking altered hemodynamics to systemic vascular remodeling.

**Figure 1:**
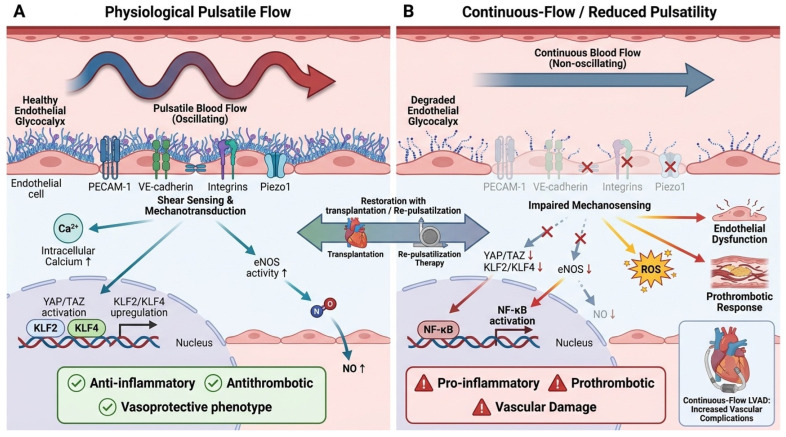
Endothelial mechanotransduction pathways under physiological pulsatile flow and continuous-flow circulation. Arrow explanations: Red and blue wavy arrows indicate pulsatile or continuous blood flow, respectively. Solid arrows indicate established biological pathways or downstream signaling events. Green arrows indicate restoration or improvement of physiological signaling following heart transplantation or re-pulsatilization therapy. Gray arrows indicate attenuation or suppression of mechanotransduction pathways. Red arrows indicate activation of pathological responses or disease progression. Yellow arrows indicate oxidative stress generation and reactive oxygen species (ROS)-mediated injury. Upward arrows (↑) denote increased activity or expression, whereas downward arrows (↓) denote decreased activity or expression. Red crosses (×) indicate impaired or inhibited mechanosensing pathways. Abbreviations: Ca^2+^, calcium ions; eNOS, endothelial nitric oxide synthase; KLF2, Krüppel-like factor 2; KLF4, Krüppel-like factor 4; NF-κB, nuclear factor kappa B; NO, nitric oxide; PECAM-1, platelet endothelial cell adhesion molecule-1; ROS, reactive oxygen species; VE-cadherin, vascular endothelial cadherin; YAP, Yes-associated protein; TAZ, transcriptional coactivator with PDZ-binding motif. Created with www.figurelabs.ai by Urbanowicz, T. (2026, ID: FL-PUB-20260603-376IAE).

Accumulating evidence suggests that the biological consequences of pulsatility extend far beyond hemodynamic regulation and involve direct modulation of endothelial mechanotransduction. Under physiological conditions, pulsatile shear stress is sensed by a coordinated mechanosensory apparatus comprising the endothelial glycocalyx, PECAM-1, VE-cadherin complexes, integrins, and mechanosensitive ion channels such as Piezo1 [94]. These signals converge on transcriptional regulators, including YAP/TAZ, KLF2, and KLF4, ultimately promoting endothelial nitric oxide synthase activation and maintenance of vascular homeostasis. In contrast, chronic exposure to continuous-flow circulation disrupts these adaptive signaling pathways, resulting in impaired nitric oxide bioavailability, inflammatory activation, oxidative stress, and endothelial dysfunction. The proposed mechanistic framework linking pulsatile hemodynamics to endothelial phenotype is summarized in Figure 1.

Biomarkers of glycocalyx injury, including syndecan-1 and heparan sulfate fragments, have emerged as potential indicators of endothelial damage and adverse outcomes [95,96]. Future studies should determine whether glycocalyx-derived biomarkers can identify patients at increased risk for bleeding, thrombosis, or microvascular dysfunction during long-term LVAD support.

### 2.2. Oxidative Stress, Mitochondrial Remodeling, and Cellular Energetics

The absence of physiological pulsatility is associated with profound disturbances in redox homeostasis [97]. Endothelial cells exposed to altered shear environments exhibit increased generation of reactive oxygen species through activation of NADPH oxidases, mitochondrial dysfunction, and uncoupling of eNOS [98].

Oxidative stress contributes to accelerated endothelial aging by inducing DNA damage, telomere shortening, and activation of cellular senescence pathways [99,100]. Senescent endothelial cells acquire a pro-inflammatory secretory phenotype characterized by increased secretion of interleukin-6, tumor necrosis factor-α, and matrix metalloproteinases.

Mitochondrial dysfunction further amplifies these processes through impaired oxidative phosphorylation and reduced ATP generation. The resulting energy imbalance affects vascular smooth muscle function, endothelial repair mechanisms, and microvascular integrity. Collectively, these alterations suggest that chronic non-pulsatile circulation may accelerate vascular aging through mechanisms extending beyond conventional cardiovascular risk factors.

#### Mitochondrial Mechanobiology and Cellular Energetics

Mitochondria are increasingly recognized as mechanosensitive organelles capable of responding directly to alterations in cellular biomechanics. Emerging evidence suggests that changes in shear stress influence mitochondrial dynamics, including fission, fusion, biogenesis, and mitophagy [101,102]. Under physiological pulsatile conditions, mitochondrial function is optimized to support endothelial nitric oxide production and redox homeostasis. In contrast, chronic exposure to continuous-flow circulation may promote mitochondrial fragmentation, impair oxidative phosphorylation, and generate excessive reactive oxygen species [103].

The concept of mitochondrial mechanobiology introduces a novel perspective on LVAD-associated vascular dysfunction. Rather than representing passive consequences of oxidative stress, mitochondrial abnormalities may constitute primary responses to altered biomechanical signaling. Restoration of pulsatile circulation following transplantation could therefore contribute to vascular recovery by normalizing mitochondrial dynamics and cellular energy metabolism.

### 2.3. Hemostasis, Acquired Von Willebrand Syndrome, and Angiogenic Dysregulation

Among the most distinctive biological consequences of CF-LVAD support is disruption of normal hemostatic regulation. High shear forces generated within contemporary pumps induce conformational changes in von Willebrand factor (vWF), exposing cleavage sites for ADAMTS13 and resulting in progressive depletion of high-molecular-weight multimers [104].

This acquired von Willebrand syndrome contributes directly to impaired platelet adhesion and increased bleeding susceptibility [105]. However, recent evidence suggests that vWF degradation also influences angiogenic signaling pathways. Loss of vWF-mediated regulation promotes excessive activation of vascular endothelial growth factor (VEGF), angiopoietin-2, and hypoxia-inducible factor-1α [106,107]. Consequently, patients supported with CF-LVADs frequently develop gastrointestinal angiodysplasia and pathological neovascularization [108]. These observations indicate that acquired von Willebrand syndrome is not merely a coagulation disorder but rather a manifestation of broader vascular remodeling induced by altered hemodynamic forces. Clinically, acquired von Willebrand syndrome develops in the majority of patients supported with contemporary continuous-flow devices and represents one of the most robust biological signatures of chronic exposure to non-physiological shear stress [109,110].

## 3. Systemic Organ Remodeling Under Continuous-Flow Circulation

### 3.1. Neurohormonal Activation and Autonomic Dysregulation

Although CF-LVAD implantation improves cardiac output and reduces ventricular filling pressures, complete normalization of neurohormonal signaling rarely occurs. Persistent activation of the renin–angiotensin–aldosterone system and sympathetic nervous system remains detectable in many patients despite successful mechanical unloading [111]. Reduced pulse pressure may impair baroreceptor function, leading to abnormal autonomic feedback and altered cardiovascular reflexes in LVAD patients [112]. This phenomenon suggests that pulsatility itself is an essential component of neurohumoral regulation, independent of absolute blood flow. The persistence of neurohormonal activation may contribute to hypertension, vascular remodeling, and progressive end-organ dysfunction during long-term support.

Recent clinical investigations further suggest that autonomic recovery following continuous-flow LVAD implantation remains incomplete despite substantial improvements in systemic perfusion [113,114]. Persistent impairment of baroreflex sensitivity, altered heart-rate variability, and sustained sympathetic activation have been associated with hypertension, arrhythmias, endothelial dysfunction, and adverse vascular remodeling during long-term support [115]. Emerging evidence also indicates that chronic neurohormonal activation interacts closely with inflammatory signaling and endothelial mechanotransduction, providing an additional biological link between altered hemodynamics and progressive cardiovascular remodeling [83,90]. These observations support continued pharmacological modulation of neurohormonal pathways even after successful mechanical unloading.

### 3.2. Microcirculatory and End-Organ Adaptation

Macrohemodynamic normalization does not necessarily translate into restoration of microvascular physiology. Continuous-flow circulation influences capillary recruitment, oxygen extraction efficiency, and regional tissue perfusion [116]. The cerebral circulation appears particularly sensitive to reduced pulsatility. Altered cerebral autoregulation has been associated with both ischemic and hemorrhagic neurological events [117]. Similar abnormalities have been observed within renal and hepatic microvascular networks [118,119], where chronic endothelial activation may contribute to progressive structural remodeling. The discrepancy between improved systemic hemodynamics and persistent microvascular dysfunction suggests that pulsatility exerts biological effects extending beyond simple blood flow delivery.

The cerebral circulation may be particularly vulnerable to chronic pulsatility deprivation because adequate cerebral perfusion depends not only on mean arterial pressure but also on dynamic transmission of pulsatile energy throughout the cerebral microvasculature. Experimental and computational studies suggest that reduced pulsatility impairs cerebral autoregulation, disrupts endothelial nitric oxide signaling, alters neurovascular coupling, and promotes blood–brain barrier dysfunction [120,121]. These alterations may increase susceptibility to microvascular injury, white matter damage, and cerebral ischemia even in the absence of large-vessel occlusion. Stroke after continuous-flow LVAD implantation is a multifactorial complication resulting from the interaction of device-related thrombosis, anticoagulation instability, hypertension, systemic or device-related infection, and patient-specific cardiovascular and thromboembolic risk factors [122]. Nevertheless, accumulating evidence suggests that chronic loss of physiological pulsatility may constitute an additional biological contributor to neurological vulnerability by impairing adaptive cerebrovascular responses [123,124]. Although direct clinical evidence establishing reduced pulsatility as an independent predictor of stroke remains limited, preservation of physiological flow dynamics represents an increasingly recognized objective in the development of future circulatory support technologies [125,126].

### 3.3. Organ-Specific Mechanobiology: Brain–Kidney–Gut Axis

Although systemic hemodynamic parameters improve substantially after LVAD implantation, individual organs exhibit distinct responses to reduced pulsatility. The cerebral circulation relies heavily on dynamic autoregulatory mechanisms that evolved under pulsatile conditions. Alterations in pulsatile energy transmission may therefore contribute to cerebral microvascular dysfunction and increased susceptibility to neurological complications. Similarly, the renal microcirculation contains highly specialized endothelial and epithelial interfaces that are sensitive to changes in pressure and flow patterns. Within the gastrointestinal tract, chronic angiogenic activation combined with altered mucosal perfusion may promote the development of angiodysplasia and recurrent bleeding. These observations suggest that organ-specific mechanobiology may represent an important determinant of long-term outcomes during mechanical circulatory support.

Endothelial dysfunction and altered mechanotransduction originate at the molecular and cellular levels; nevertheless their consequences ultimately extend to entire organ systems [127]. Importantly, individual organs do not respond uniformly to chronic pulsatility deprivation but instead exhibit distinct patterns of adaptation determined by their microvascular architecture, metabolic requirements, and dependence on pulsatile energy transmission. The brain, kidney, liver, gastrointestinal tract, pulmonary circulation, and hematopoietic system appear particularly susceptible to alterations in shear stress signaling and endothelial homeostasis. Consequently, continuous-flow LVAD support should be viewed not only as a hemodynamic intervention but also as a systemic biological state characterized by heterogeneous organ-specific responses.

**Figure 2:**
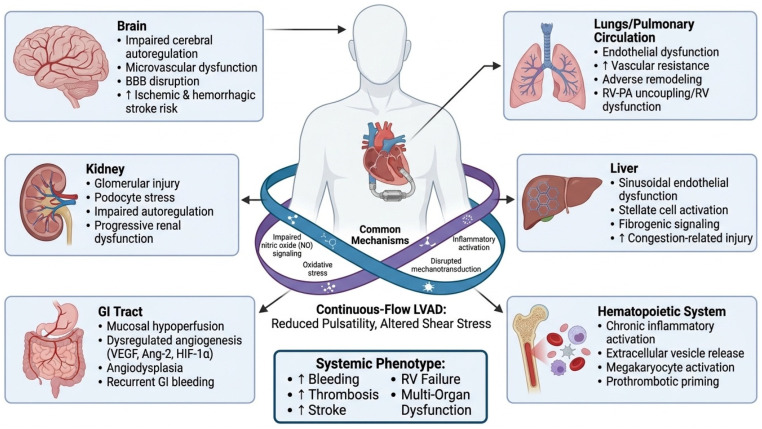
Organ-Specific Mechanobiology and Clinical Consequences of Pulsatility Deprivation During Continuous-Flow LVAD Support. Arrow explanations: Black arrows indicate organ-specific effects and systemic biological interactions associated with continuous-flow left ventricular assist device (LVAD) support. The circular blue–purple ribbon illustrates the bidirectional interplay among impaired nitric oxide (NO) signaling, oxidative stress, inflammatory activation, and disrupted mechanotransduction, which collectively drive multi-organ adaptation and injury. Upward arrows (↑) denote increased activity, expression, or risk. Abbreviations: BBB, blood–brain barrier; GI, gastrointestinal; HIF-1α, hypoxia-inducible factor-1α; LVAD, left ventricular assist device; NO, nitric oxide; ROS, reactive oxygen species; RV, right ventricle; RV-PA, right ventricle–pulmonary artery; VEGF, vascular endothelial growth factor. Created with www.figurelabs.ai by Urbanowicz, T. (2026, ID: FL-PUB-20260603-BW3AZB).

The major mechanisms linking reduced pulsatility to organ remodeling and clinical complications are summarized in Figure 2. Attention should be paid to the cerebral circulation, where impaired autoregulation, altered neurovascular coupling, and endothelial dysfunction may collectively contribute to the increased susceptibility to neurological complications observed during long-term continuous-flow support.

The presented observations suggest that the biological consequences of pulsatility deprivation extend beyond the vasculature and involve coordinated remodeling across multiple organ systems, highlighting the need for integrated molecular and systems-level approaches to understanding long-term adaptation to continuous-flow circulatory support [128].

### 3.4. Extracellular Vesicles, MicroRNAs, and Emerging Molecular Signatures

Recent advances in molecular profiling have identified extracellular vesicles and circulating microRNAs as potential mediators of vascular adaptation during mechanical circulatory support [129]. Specific microRNAs involved in angiogenesis, inflammation, fibrosis, and endothelial function demonstrate altered expression following LVAD implantation [130,131]. Similarly, extracellular vesicles released from activated endothelial cells, platelets, and leukocytes may facilitate inter-organ communication and contribute to systemic remodeling.

Transcriptomic and proteomic studies increasingly suggest that CF-LVAD support induces a unique molecular phenotype distinct from both advanced heart failure and physiological circulation [132]. These findings provide a framework for identifying biomarkers that predict bleeding, thrombosis, right ventricular failure, and adverse vascular remodeling.

High-throughput omics technologies provide unprecedented opportunities to characterize the molecular consequences of pulsatility deprivation [133]. Transcriptomic analyses [132] have demonstrated differential expression of genes involved in endothelial function, inflammation, angiogenesis, and extracellular matrix remodeling following LVAD implantation. Proteomic studies have identified alterations in coagulation factors, inflammatory mediators, oxidative stress proteins, and angiogenic regulators, while metabolomic investigations reveal disturbances in mitochondrial metabolism and redox pathways. More recently, single-cell RNA sequencing has enabled characterization of cell-specific responses within vascular tissues, revealing previously unrecognized heterogeneity in endothelial, immune, and smooth muscle cell populations [134]. Integration of transcriptomic, proteomic, metabolomic, and single-cell datasets may ultimately define a distinct molecular signature of continuous-flow circulation.

An important yet incompletely understood aspect of continuous-flow LVAD support is the marked heterogeneity of biological adaptation observed among individual patients. Clinical experience has demonstrated substantial interindividual variability in adaptation to continuous-flow LVAD support. While some recipients develop recurrent bleeding, thrombotic complications, or progressive end-organ dysfunction, others tolerate prolonged device support with relatively few major adverse events [135]. This variability likely reflects differences in genetic background, baseline endothelial reserve, inflammatory responsiveness, mitochondrial function, immunometabolic adaptation, comorbidity burden, and the duration of advanced heart failure prior to device implantation [136]. Differences in device settings, residual ventricular function, and the degree of preserved pulsatility may further modify biological responses. Identification of molecular signatures associated with favorable adaptation may ultimately facilitate personalized risk stratification and individualized optimization of long-term mechanical circulatory support.

**Figure 3:**
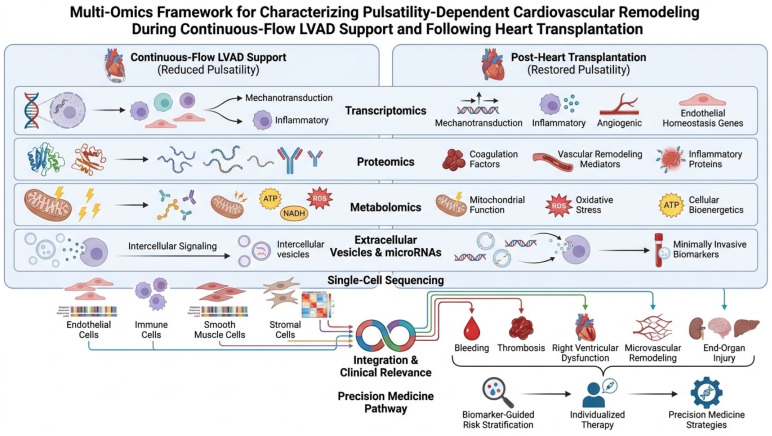
Multi-Omics Framework for Characterizing Pulsatility-Dependent Cardiovascular Remodeling During Continuous-Flow LVAD Support and Following Heart Transplantation. Arrow explanations: Black arrows indicate the direction of biological processes, molecular interactions, or progression from mechanistic alterations to clinical application. Bidirectional arrows indicate dynamic transitions or restoration of biological pathways following heart transplantation. Curved arrows denote intracellular communication, molecular trafficking, or regulatory feedback. Colored connecting lines illustrate the integration of multi-omic datasets into single-cell analysis, clinical interpretation, and precision medicine. Abbreviations: ATP, adenosine triphosphate; LVAD, left ventricular assist device; NADH, nicotinamide adenine dinucleotide (reduced form); ROS, reactive oxygen species. Created with www.figurelabs.ai by Urbanowicz, T. (2026, ID: FL-PUB-20260603-VFY0U8).

Although individual biomarkers provide valuable insights into specific biological pathways, they are unlikely to capture the complexity of cardiovascular adaptation to altered pulsatile hemodynamics. The emerging integration of transcriptomics, proteomics, metabolomics, extracellular vesicle profiling, and single-cell sequencing offers a systems-biology approach capable of characterizing pulsatility-dependent remodeling across multiple levels of biological organization. Such multi-omics strategies may facilitate identification of molecular signatures associated with endothelial dysfunction, angiogenic activation, oxidative stress, inflammation, and organ-specific adaptation during continuous-flow LVAD support. Furthermore, integration of these datasets has the potential to improve risk stratification, predict adverse events, and support development of personalized therapeutic interventions. A proposed multi-omics framework for investigating pulsatility-dependent cardiovascular remodeling is presented in Figure 3.

A practical implementation of this framework would involve serial collection of blood samples before LVAD implantation, during mechanical support, and following heart transplantation. Transcriptomic, proteomic, metabolomic, extracellular vesicle, and single-cell sequencing data could then be integrated using systems biology and machine-learning approaches to identify molecular signatures associated with bleeding, thrombosis, right ventricular failure, neurological complications, or vascular recovery. Finally, these integrated molecular profiles could be linked to imaging, hemodynamic measurements, and long-term clinical outcomes to generate predictive models that support precision management of patients undergoing advanced mechanical circulatory support.

Interpretation of multi-omics datasets remains challenging because molecular signatures may be influenced by device type, duration of support, comorbidities, immunosuppressive therapy, and concomitant pharmacological treatment. Consequently, future investigations will require standardized sampling strategies and prospective validation cohorts.

## 4. Restoration of Pulsatility After Heart Transplantation

### 4.1. Reversal of Endothelial Dysfunction Following Restoration of Physiological Hemodynamics

Heart transplantation restores physiological pulse pressure and normal pressure wave propagation throughout the arterial tree. This re-establishment of pulsatile shear stress reactivates endothelial signaling pathways that are suppressed during advanced heart failure and continuous-flow support. Improved nitric oxide bioavailability, enhanced KLF2 expression, reduced endothelin-1 production, and normalization of endothelial responsiveness collectively contribute to restoration of vascular homeostasis [137]. Clinical studies [138] demonstrating improved flow-mediated dilation following transplantation support the concept that endothelial dysfunction is, at least partially, reversible. These observations reinforce the notion that endothelial phenotype is dynamically regulated by hemodynamic forces rather than irreversibly determined by structural vascular disease.

#### Vascular Remodeling and Microvascular Recovery Following Heart Transplantation

Restoration of physiological pulsatility following heart transplantation influences not only endothelial signaling but also vascular structure and microvascular function. In advanced heart failure, prolonged exposure to low cardiac output, neurohormonal activation, systemic inflammation, and endothelial dysfunction promote arterial stiffening, impaired vasodilatory capacity, and abnormalities of microvascular autoregulation [139,140]. Although these alterations may develop gradually over years, several studies suggest that at least part of this vascular phenotype remains reversible after successful transplantation [141].

The re-establishment of physiological pulse pressure restores cyclic stretch and oscillatory shear stress throughout the arterial tree, thereby reactivating mechanosensitive pathways involved in vascular homeostasis [85]. Improved nitric oxide bioavailability and reduced vasoconstrictor signaling contribute to enhanced endothelial responsiveness and more effective regulation of vascular tone [142]. Clinical investigations have demonstrated improvements in flow-mediated dilation, arterial compliance, and peripheral vascular function during the months following transplantation, supporting the concept that vascular adaptation remains a dynamic process rather than a fixed consequence of chronic heart failure.

Microvascular recovery may be particularly important. Adequate tissue perfusion depends not only on the restoration of cardiac output but also on the ability of resistance vessels and capillary networks to appropriately distribute blood flow according to metabolic demand [143]. Experimental and clinical observations indicate that normalization of pulsatile hemodynamics may improve capillary recruitment, endothelial–microvascular coupling, and tissue oxygen extraction. These effects could contribute to the progressive recovery of renal, hepatic, skeletal muscle, and cerebral function, which is frequently observed after transplantation.

Nevertheless, vascular recovery following transplantation is often incomplete. Persistent endothelial dysfunction, chronic inflammation, metabolic disturbances, and exposure to immunosuppressive therapy may limit restoration of normal vascular biology. Furthermore, the development of cardiac allograft vasculopathy demonstrates that normalization of pulsatility alone does not guarantee long-term vascular health. These observations suggest that pulsatile hemodynamics are necessary but not sufficient for full vascular recovery, and that vascular remodeling after transplantation reflects the interaction between restored biomechanical signaling and ongoing immune-mediated processes.

Available clinical evidence suggests that recovery of endothelial function begins within weeks after successful heart transplantation, whereas improvements in arterial compliance, microvascular function, and vascular remodeling generally evolve over several months and may continue throughout the first postoperative year [144,145]. However, the rate and extent of vascular recovery vary considerably among individual recipients. The rate and extent of endothelial recovery after heart transplantation are likely influenced by advanced recipient age, pre-existing vascular disease, diabetes mellitus, chronic kidney disease, the severity and duration of pre-transplant heart failure, and the vascular effects of long-term immunosuppressive therapy [146,147]. Although sex- and race-specific data remain limited, current evidence suggests that baseline cardiovascular risk profiles and comorbidity burden exert a greater influence on vascular recovery than demographic characteristics alone [148,149]. Prospective studies incorporating standardized vascular phenotyping will be required to clarify these potential sources of biological variability.

From a mechanobiological perspective, heart transplantation offers a unique human model for investigating the reversibility of pulsatility-dependent cardiovascular remodeling. Understanding which vascular abnormalities recover after restoration of physiological flow and which persist despite normalization of hemodynamics may help identify biological pathways directly regulated by pulsatility and distinguish them from mechanisms driven primarily by inflammation, immune activation, or irreversible structural injury.

### 4.2. Immune–Vascular Crosstalk in the Transplanted Circulation

Unlike LVAD support, heart transplantation introduces complex immunological interactions. Immunosuppressive therapy, alloimmune responses, and chronic rejection pathways create a unique biological environment that coexists with restored pulsatility. Although inflammatory biomarkers generally decline following transplantation, immune-mediated endothelial injury remains an important determinant of long-term outcomes [150]. Endothelial cells function as active participants in alloimmune recognition, linking vascular biology and transplant immunology [151]. The interaction between restored pulsatile signaling and chronic immune activation represents an underexplored area with substantial translational significance.

### 4.3. Immunometabolic Remodeling During Pulsatility Loss and Restoration

Beyond vascular biology, altered hemodynamics influence immune cell metabolism and function. Recent publications [152] indicate that mechanical forces regulate macrophage polarization, T-cell activation, and innate immune signaling through pathways involving mTOR, HIF-1α, AMPK, and mitochondrial metabolism. Continuous-flow support may promote a persistent pro-inflammatory immunometabolic state characterized by enhanced glycolysis and oxidative stress, whereas restoration of physiological pulsatility following transplantation may partially reverse these abnormalities. The interaction between mechanotransduction and immunometabolism represents an emerging field with significant implications for vascular inflammation, adverse remodeling, and long-term device-related complications.

#### 4.3.1. Cardiac Allograft Vasculopathy: When Pulsatility Restoration Is Not Enough

Although heart transplantation restores physiological pulsatile hemodynamics and re-establishes many endothelial signaling pathways disrupted during advanced heart failure and continuous-flow mechanical circulatory support, vascular homeostasis is not completely normalized. The most compelling evidence for this limitation is provided by cardiac allograft vasculopathy (CAV), a unique form of diffuse and progressive vascular disease that remains a leading cause of long-term graft dysfunction and mortality following transplantation [153,154].

Unlike conventional atherosclerosis, which typically involves focal plaque formation within epicardial coronary arteries, CAV is characterized by concentric intimal hyperplasia, diffuse luminal narrowing, microvascular dysfunction, and progressive impairment of coronary vasodilatory capacity [155]. The pathogenesis of CAV is multifactorial and involves a complex interplay between alloimmune injury, chronic inflammation, endothelial activation, oxidative stress, and maladaptive vascular remodeling [156]. Endothelial cells occupy a central position within this process, functioning not only as passive targets of immune-mediated injury but also as active participants in antigen presentation, leukocyte recruitment, cytokine production, and vascular remodeling [151,157].

Restoration of physiological pulsatility improves endothelial mechanotransduction and vascular homeostasis; however, these beneficial effects occur within a complex immunological environment in which alloimmune responses, chronic inflammation, donor-specific antibodies, complement activation, and immunosuppressive therapy remain the principal determinants of cardiac allograft vasculopathy [158]. Consequently, pulsatility should be regarded as a permissive modifier of vascular biology rather than the primary pathogenic driver of CAV.

Nevertheless, these beneficial effects coexist with persistent immunological stressors that may override or counterbalance the protective influence of pulsatile hemodynamics. Donor-specific antibodies, T-cell-mediated immune responses, complement activation, and chronic low-grade inflammation contribute to ongoing endothelial injury despite normalization of pulse pressure and shear stress patterns [159,160]. As a consequence, endothelial dysfunction may persist even in the presence of restored physiological flow dynamics.

Recent studies [161] further indicate that immune activation and mechanotransduction are not independent biological processes but rather interact through shared signaling pathways involving NF-κB, reactive oxygen species, mitochondrial dysfunction, and inflammatory cytokine networks. These observations suggest that vascular adaptation following transplantation is determined by the balance between restorative pulsatile signaling and persistent immune-mediated injury. In this context, CAV may be viewed as a biological counterexample demonstrating that restoration of pulsatility alone is insufficient to ensure long-term vascular health when other pathological drivers of endothelial dysfunction remain active.

The coexistence of physiological pulsatility and chronic vascular injury following transplantation provides an important conceptual complement to the vascular abnormalities observed during continuous-flow LVAD support. Whereas LVAD-associated complications arise predominantly in the setting of impaired mechanotransduction and pulsatility deprivation, CAV develops despite restoration of physiological hemodynamics. Together, these observations support a broader paradigm in which pulsatility functions as a critical but not exclusive regulator of cardiovascular biology. Long-term vascular homeostasis appears to require both preservation of physiological biomechanical signaling and effective control of inflammatory and immune-mediated pathways. Understanding the interactions among these processes may provide novel therapeutic opportunities to improve outcomes after both mechanical circulatory support and heart transplantation.

#### 4.3.2. Restoration of Arterial Stiffness and Vascular Aging Pathways After Heart Transplantation

The biological consequences of heart transplantation extend beyond restoration of cardiac output and normalization of endothelial signaling. Increasing evidence suggests that recovery of physiological pulsatility may also influence processes associated with vascular aging, arterial stiffness, and large-vessel remodeling. These adaptations are of particular interest because patients with advanced heart failure frequently exhibit an accelerated vascular aging phenotype characterized by endothelial dysfunction, increased arterial stiffness, oxidative stress, chronic inflammation, and impaired vasodilatory reserve [72,162].

Under physiological conditions, large arteries serve not only as passive conduits for blood flow but also as dynamic biomechanical structures that buffer pulsatile energy generated by ventricular contraction. The elastic properties of the aorta and major arteries contribute to maintenance of normal pulse wave propagation, ventricular–vascular coupling, and microvascular protection. In advanced heart failure, these physiological relationships become progressively disturbed. Reduced stroke volume, neurohormonal activation, oxidative stress, and chronic systemic inflammation promote structural alterations within the vascular wall, including collagen accumulation, elastin fragmentation, smooth muscle cell dysfunction, and extracellular matrix remodeling [163]. Collectively, these changes contribute to increased arterial stiffness and impaired vascular compliance.

Heart transplantation restores physiological pulsatile flow and re-establishes the cyclical mechanical forces that normally regulate vascular structure and function. Several studies have demonstrated improvements in arterial compliance and endothelial-dependent vasodilation following transplantation, suggesting that at least part of the vascular dysfunction associated with end-stage heart failure remains reversible [164]. Recovery of pulsatile hemodynamics may facilitate normalization of nitric oxide signaling, reduction in oxidative stress, and restoration of vascular smooth muscle responsiveness, thereby improving the functional properties of the arterial wall [165].

The relationship between pulsatility and vascular aging is increasingly supported by experimental evidence indicating that biomechanical forces directly regulate molecular pathways involved in cellular senescence. Endothelial cells exposed to physiological pulsatile shear stress demonstrate enhanced activation of protective transcriptional programs involving KLF2, KLF4, and endothelial nitric oxide synthase, whereas disturbed flow patterns promote oxidative injury, inflammatory signaling, and senescence-associated phenotypes [166]. These observations raise the possibility that restoration of physiological pulsatility after transplantation may partially reverse vascular aging pathways that become activated during advanced heart failure and chronic circulatory dysfunction.

Mitochondrial function may represent an important mechanistic link between pulsatile hemodynamics and vascular aging [167]. Aging-associated vascular dysfunction has been closely linked to mitochondrial DNA damage, impaired oxidative phosphorylation, and excessive reactive oxygen species generation. Restoration of physiological biomechanical signaling may improve mitochondrial homeostasis through mechanisms involving nitric oxide bioavailability, AMPK activation, and improved endothelial metabolism. Although direct evidence in transplant recipients remains limited, these pathways provide a biologically plausible explanation for the progressive improvement in vascular function observed following successful transplantation.

Nevertheless, recovery of vascular biology after transplantation is rarely complete. Persistent exposure to cardiovascular risk factors, chronic kidney disease, metabolic abnormalities, and long-term immunosuppressive therapy may continue to promote arterial stiffening and endothelial dysfunction despite restoration of physiological pulsatility. Calcineurin inhibitors, in particular, have been associated with hypertension, oxidative stress, and adverse vascular remodeling, potentially counteracting some of the beneficial effects of restored hemodynamics [168]. Consequently, vascular aging after transplantation likely reflects the balance between regenerative influences associated with physiological pulsatility and ongoing pathological stimuli related to immunological and metabolic factors.

The coexistence of vascular recovery and persistent vascular injury highlights an important concept emerging from contemporary mechanobiology. Pulsatility appears to contribute substantially to maintenance of vascular homeostasis, yet restoration of physiological pulse pressure alone is insufficient to fully reverse established biological aging processes. Instead, long-term vascular health after transplantation is determined by the interaction among biomechanical signaling, immune regulation, metabolic homeostasis, and environmental risk factors. Future studies combining measurements of arterial stiffness, pulse wave velocity, endothelial function, and molecular markers of cellular senescence may help clarify the extent to which restoration of pulsatility modifies vascular aging trajectories in transplant recipients.

From a broader perspective, heart transplantation provides a unique clinical model for investigating the reversibility of cardiovascular aging. Comparison of patients exposed to prolonged periods of reduced pulsatility during advanced heart failure or mechanical circulatory support with those undergoing restoration of physiological pulsatile circulation may provide valuable insights into the role of biomechanical forces in vascular longevity. Understanding these mechanisms could ultimately inform the development of novel therapeutic strategies aimed at preserving vascular function not only after transplantation but also during long-term mechanical circulatory support.

### 4.4. Comparative Molecular Framework: Pulsatility Deprivation Versus Pulsatility Restoration

Continuous-flow LVAD support and heart transplantation should not be viewed solely as alternative therapeutic strategies. Rather, they constitute two opposing biological states characterized by chronic pulsatility deprivation and pulsatility restoration. Although individual molecular pathways provide valuable mechanistic insights, the biological effects of pulsatility extend across multiple levels of physiological organization. Hemodynamic forces are initially sensed by mechanotransduction complexes at the endothelial surface but subsequently influence cellular metabolism, tissue architecture, organ function, and ultimately clinical outcomes. Consequently, pulsatility may be viewed as a systems-level regulator operating across interconnected biological hierarchies. A conceptual framework illustrating the multi-scale consequences of pulsatility deprivation and restoration is proposed in Table 3.

Loss of pulsatility is associated with impaired mechanotransduction, oxidative stress, inflammation, angiogenic dysregulation, acquired von Willebrand syndrome, and microvascular dysfunction. Restoration of pulsatility promotes endothelial recovery, normalization of vascular signaling, and improved organ homeostasis. This comparative framework provides a novel conceptual model for understanding the biological importance of pulsatile blood flow and highlights mechanotransduction as a potentially emerging determinant of long-term cardiovascular health.

The findings discussed throughout this review indicate that pulsatility influences cardiovascular biology through a highly interconnected network of mechanosensitive, inflammatory, metabolic, angiogenic, and hemostatic pathways. Importantly, these molecular systems do not function independently but instead form an integrated signaling architecture that determines endothelial phenotype, vascular integrity, microcirculatory function, and end-organ adaptation. Continuous-flow LVAD support and heart transplantation therefore represent contrasting biological states associated with differential activation of these pathways. Identification of the principal molecular mediators involved in pulsatility-dependent signaling may facilitate the development of targeted therapeutic interventions and next-generation circulatory support technologies. The major pathways currently implicated in hemodynamic mechanobiology and their potential translational significance are summarized in Table 4.

**Figure 4:**
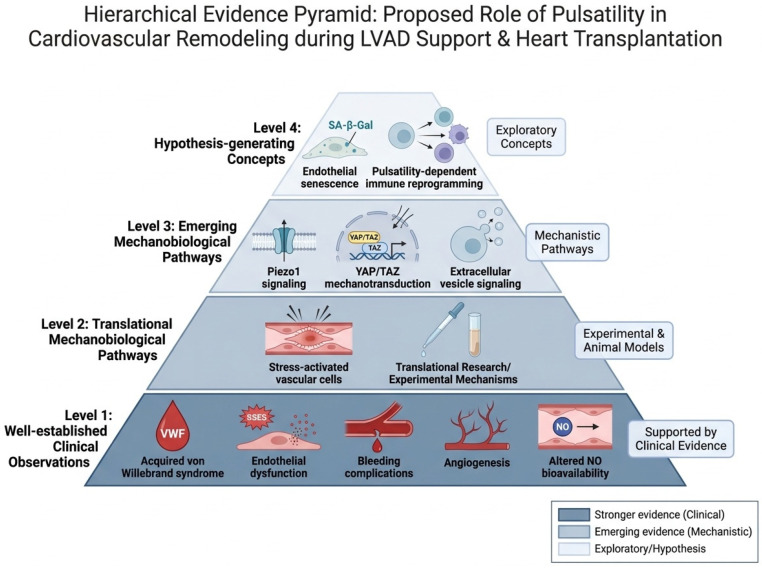
Evidence pyramid of pulsatility-dependent biological pathways during continuous-flow LVAD support and following heart transplantation. Arrow explanations: Black arrows indicate biological progression, mechanistic relationships, or transitions between consecutive levels of evidence. Diverging arrows indicate the emergence of multiple downstream biological pathways or cellular responses from a common upstream mechanism. The right-sided boxes denote the predominant type of supporting evidence at each hierarchical level, ranging from established clinical observations to exploratory hypotheses. Abbreviations: NO, nitric oxide; SA-β-Gal, senescence-associated β-galactosidase; YAP, Yes-associated protein; TAZ, transcriptional coactivator with PDZ-binding motif; vWF, von Willebrand factor. Created with www.figurelabs.ai by Urbanowicz, T. (2026, ID: FL-PUB-20260718-QCQGF1).

As shown in Table 3, mechanotransduction, endothelial homeostasis, oxidative stress, angiogenic regulation, immunometabolic adaptation, and hemostatic remodeling represent interconnected responses to altered hemodynamic stimuli. We have presented the numerous molecular pathways that have been implicated in the biological response to altered pulsatile hemodynamics; the strength of supporting evidence varies considerably among proposed mechanisms. Certain processes, including acquired von Willebrand syndrome, endothelial dysfunction, and angiogenic dysregulation, are supported by extensive clinical observations and translational investigations in LVAD recipients. In contrast, pathways involving glycocalyx remodeling, Piezo1-mediated mechanotransduction, YAP/TAZ signaling, extracellular vesicle biology, immunometabolic adaptation, and multi-omics signatures remain incompletely validated in human studies and are supported predominantly by experimental or hypothesis-generating evidence. Recognition of these differences is essential for interpreting current knowledge and for identifying priorities for future research. To provide a conceptual overview of the relative maturity of the available evidence, the principal pulsatility-dependent biological pathways are organized according to their current level of clinical and mechanistic validation in Figure 4.

**Figure 5:**
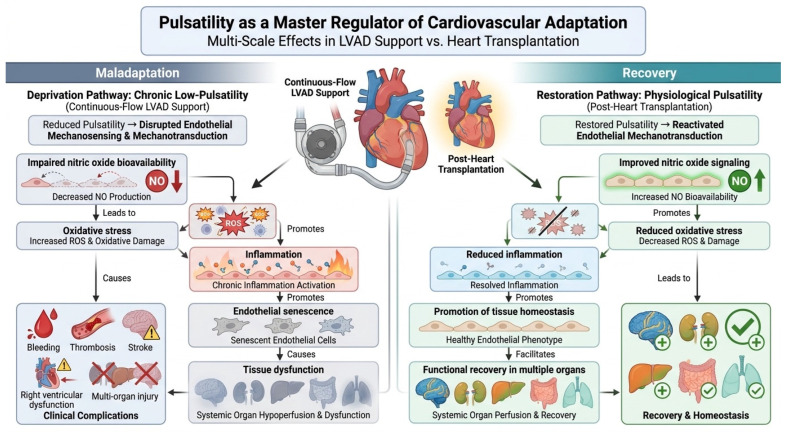
Multi-Scale Hierarchical Model of Pulsatility-Dependent Cardiovascular Regulation During Continuous-Flow LVAD Support and Following Heart Transplantation. Arrow explanations: Solid black arrows indicate the direction of biological signaling, mechanistic progression, or cause-and-effect relationships. Green arrows indicate restoration of physiological pathways and recovery of endothelial homeostasis following heart transplantation. Downward arrows (↓) denote reduced activity, expression, or biological function, whereas upward arrows (↑) denote increased activity or restoration of physiological processes. The central black arrows illustrate the transition from continuous-flow LVAD support to heart transplantation and the corresponding shift from maladaptive to recovery-associated biological pathways. Plus symbols (+) indicate improvement or restoration of organ function, whereas warning symbols (⚠) indicate increased risk of clinical complications. Abbreviations: LVAD, left ventricular assist device; NO, nitric oxide; ROS, reactive oxygen species. Created with www.figurelabs.ai by Urbanowicz, T. (2026, ID: FL-PUB-20260718-IJB04S).

The strength of evidence varies among pathways; this framework supports the concept that pulsatility may influence cardiovascular adaptation across multiple biological scales. In this conceptual framework, continuous-flow LVAD support and heart transplantation represent opposing biological trajectories characterized by progressive adaptation to pulsatility deprivation and progressive recovery following restoration of physiological pulsatility, respectively. The proposed multi-scale model of pulsatility-dependent cardiovascular regulation is illustrated in Figure 5.

An important unresolved question is whether the biological alterations observed during continuous-flow LVAD support are attributable primarily to pulsatility deprivation itself or reflect the combined influence of multiple concurrent factors, including advanced heart failure, chronic inflammation, neurohormonal activation, device-related shear stress, anticoagulation therapy, and patient-specific comorbidities. Although growing evidence supports a mechanistic role for altered pulsatile hemodynamics in endothelial dysfunction, angiogenic activation, oxidative stress, and microvascular remodeling, direct causal relationships remain difficult to establish in human studies. Furthermore, many molecular pathways implicated in pulsatility-dependent adaptation, including Piezo1 signaling, YAP/TAZ regulation, glycocalyx remodeling, and immunometabolic reprogramming, have been characterized predominantly in experimental models rather than in prospective investigations of LVAD recipients. It is therefore likely that pulsatility functions as one component of a broader mechanobiological network rather than as an isolated determinant of vascular homeostasis. Future studies integrating hemodynamic measurements with molecular phenotyping and longitudinal clinical outcomes will be essential for distinguishing pulsatility-specific effects from other contributors to cardiovascular remodeling and for defining the relative importance of biomechanical signaling within the complex biological environment of mechanical circulatory support.

#### 4.4.1. Establishing Causality: Is Pulsatility the Driver or a Marker?

Although associations between reduced pulsatility and adverse biological remodeling are increasingly recognized, the extent to which pulsatility directly causes these effects remains uncertain. Reduced pulsatility frequently coexists with advanced heart failure, systemic inflammation, altered neurohormonal signaling, anticoagulation therapy, device-related shear stress, and multiple comorbid conditions. Consequently, pulsatility may function both as a biological regulator and as a surrogate marker of broader cardiovascular adaptation. Future investigations combining standardized pulsatility metrics with longitudinal molecular profiling and mechanistic intervention studies will be required to determine whether restoration of pulsatile hemodynamics directly modifies vascular biology or merely accompanies recovery induced by other factors.

#### 4.4.2. Strength of Current Evidence

Although the conceptual framework proposed in this review integrates multiple biological pathways, the strength of the supporting evidence varies substantially across mechanisms. Associations involving acquired von Willebrand syndrome, endothelial dysfunction, and angiogenic dysregulation are supported by extensive clinical observations in LVAD recipients. In contrast, the proposed contributions of glycocalyx remodeling, Piezo1 signaling, YAP/TAZ mechanotransduction, and immunometabolic adaptation are derived predominantly from experimental and translational studies. Consequently, these pathways should be regarded as biologically plausible but incompletely validated mechanisms requiring prospective investigation. Recognition of these differences is essential for interpreting the current state of knowledge and for prioritizing future mechanistic research.

Although numerous molecular pathways have been linked to altered pulsatile hemodynamics, the available evidence is not equally robust across all proposed mechanisms. Clinical observations strongly support the roles of acquired von Willebrand syndrome, endothelial dysfunction, and angiogenic activation during continuous-flow LVAD support. By contrast, pathways involving glycocalyx injury, Piezo1 and YAP/TAZ signaling, immunometabolic remodeling, and vascular aging remain supported primarily by experimental data and indirect clinical observations. A summary of the current evidence supporting individual pulsatility-dependent mechanisms is presented in Table 5.

#### 4.4.3. Contradictory and Negative Evidence

Though a growing body of literature supports the concept that pulsatility influences vascular biology and cardiovascular adaptation, several observations challenge the interpretation that pulsatility deprivation alone is responsible for the complications observed during continuous-flow LVAD support [201,202]. Recognition of these findings is essential for maintaining a balanced perspective and for defining the current limits of mechanobiological evidence.

First, many biological abnormalities attributed to reduced pulsatility may also be explained by factors that coexist with continuous-flow support. Advanced heart failure itself is characterized by endothelial dysfunction, chronic inflammation, oxidative stress, neurohormonal activation, mitochondrial impairment, and microvascular abnormalities. Consequently, some molecular alterations observed after LVAD implantation may represent persistence of pre-existing disease processes rather than direct consequences of altered pulsatile hemodynamics. Distinguishing between residual heart failure biology and pulsatility-dependent remodeling remains a major challenge in clinical investigations.

Second, several studies have demonstrated substantial clinical improvement despite persistent reductions in physiological pulsatility [203]. Contemporary continuous-flow LVADs provide durable circulatory support, improve functional capacity, enhance end-organ perfusion, and prolong survival compared with historical pulsatile devices and medical therapy alone. These observations indicate that restoration of physiological pulse pressure is not required for many aspects of cardiovascular recovery. The existence of long-term LVAD recipients with relatively few vascular complications further suggests that adaptation to reduced pulsatility may be more heterogeneous than initially assumed.

Additional uncertainty arises from studies evaluating artificial pulsatility and variable-speed pump algorithms. Although experimental investigations have shown favorable effects on hemodynamic parameters and endothelial signaling, clinical studies have not consistently demonstrated reductions in bleeding, thrombosis, stroke, or mortality [204]. The absence of definitive clinical benefit raises the possibility that the magnitude, frequency, or biological relevance of currently generated pulsatile signals may be insufficient to reproduce native cardiovascular physiology. Alternatively, pulsatility may represent only one component of a more complex mechanobiological environment.

Evidence from heart transplantation also provides important counterarguments. Restoration of physiological pulsatile circulation does not completely normalize vascular biology [205,206]. Cardiac allograft vasculopathy, endothelial dysfunction, microvascular abnormalities, and chronic inflammatory activation may develop despite restoration of physiological pulse pressure and shear stress patterns [207]. These observations indicate that pulsatility alone cannot fully determine vascular homeostasis and that immune-mediated, metabolic, and inflammatory pathways may exert equally important influences on long-term vascular remodeling.

Furthermore, several mechanistic pathways discussed throughout this review—including glycocalyx remodeling, Piezo1 signaling, YAP/TAZ regulation, extracellular vesicle biology, and immunometabolic adaptation—have been investigated predominantly in experimental systems [60,179]. Direct human evidence linking these pathways to pulsatility deprivation during long-term LVAD support remains limited. As a result, many proposed mechanisms should currently be considered biologically plausible hypotheses rather than definitively established causal pathways.

Taken together, the available data suggest that pulsatility should not be viewed as the sole determinant of cardiovascular adaptation. Instead, pulsatility likely interacts with multiple concurrent factors, including device-related shear stress, residual heart failure biology, neurohormonal activation, inflammation, anticoagulation therapy, and patient-specific characteristics [201,208]. The most accurate interpretation of current evidence may therefore be that pulsatility functions as an important component of a broader mechanobiological network regulating vascular homeostasis. Future prospective studies integrating quantitative pulsatility measurements with molecular phenotyping and clinical outcomes will be required to determine the relative contribution of pulsatile hemodynamics to cardiovascular remodeling during mechanical circulatory support and following heart transplantation.

### 4.5. Future Directions, Limitations

#### 4.5.1. Current Knowledge Gaps

Most available evidence originates from observational studies with heterogeneous patient populations, device generations, and follow-up durations. Mechanistic investigations directly comparing CF-LVAD recipients and transplant recipients remain scarce. Consequently, causal relationships between pulsatility and molecular remodeling are often inferred rather than definitively demonstrated.

#### 4.5.2. Limitations of Existing Research

A major limitation of current literature is the absence of standardized measures of pulsatility and vascular biological responses. Furthermore, many studies focus on clinical outcomes without integrating molecular, transcriptomic, proteomic, and metabolomic data. This fragmentation limits understanding of the biological pathways linking altered hemodynamics to clinical complications.

**Figure 6:**
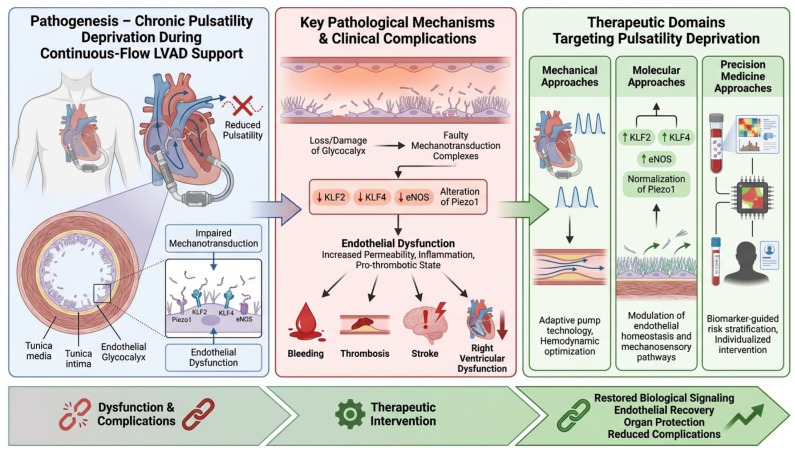
Pulsatility as a Therapeutic Target: From Mechanobiological Dysfunction to Biological Restoration. Arrow explanations: Solid black arrows indicate the direction of biological signaling, mechanistic progression, or cause-and-effect relationships. Large blue and green arrows represent progression from disease mechanisms to therapeutic intervention and biological recovery. Green arrows indicate restoration or enhancement of physiological pathways following therapeutic intervention. Upward arrows (↑) denote increased expression, activation, or biological activity, whereas downward arrows (↓) denote decreased expression, activity, or impaired physiological function. Red cross (×) indicates loss or absence of physiological pulsatility. The broken chain symbol indicates interruption of pathological processes, whereas the intact green chain symbolizes restoration of biological continuity and tissue homeostasis. Abbreviations: eNOS, endothelial nitric oxide synthase; KLF2, Krüppel-like factor 2; KLF4, Krüppel-like factor 4; LVAD, left ventricular assist device; NO, nitric oxide; Piezo1, piezo-type mechanosensitive ion channel component 1. Created with www.figurelabs.ai by Urbanowicz, T. (2026, ID: FL-PUB-20260603-T6DPNP).

The evidence discussed throughout this review suggests that many complications observed during long-term continuous-flow LVAD support arise not solely from altered hemodynamics but from disruption of pulsatility-dependent biological signaling. Consequently, future therapeutic strategies should move beyond restoration of systemic blood flow and aim to preserve or re-establish physiological mechanotransduction pathways. Such approaches may include development of next-generation devices capable of generating adaptive pulsatility, pharmacological interventions targeting endothelial dysfunction and glycocalyx injury, modulation of mechanosensitive signaling pathways, and implementation of biomarker-guided precision medicine strategies. Beyond advances in pump engineering, regenerative biomaterials may further improve functional recovery following heart transplantation. Conductive hydrogels, including poly(3,4-ethylenedioxythiophene):poly(styrenesulfonate) (PEDOT:PSS)-based platforms, have demonstrated favorable electrical conductivity, mechanical compliance, and excellent cytocompatibility in experimental cardiac models [209,210,211]. By improving electrical signal propagation, supporting cardiomyocyte organization, and promoting integration with host myocardium, these biomaterials may facilitate restoration of myocardial electromechanical function while reducing arrhythmogenic substrate formation [212]. Although clinical translation remains in its early stages, integration of smart biomaterials with regenerative and bioelectronic strategies represents a promising future direction for improving long-term graft performance after heart transplantation. Collectively, these interventions share a common objective: restoration of vascular homeostasis through recovery of pulsatility-dependent biological regulation. The proposed translational framework linking mechanobiological dysfunction to biological restoration is summarized in Figure 6.

The presented Figure 6 highlights the future paradigm shift from flow replacement alone toward restoration of pulsatility-dependent biological regulation as a central objective of mechanical circulatory support.

#### 4.5.3. Translational Implications

Recognition of pulsatility as a biological regulator rather than a purely hemodynamic phenomenon has important therapeutic implications. Future LVAD technologies may incorporate adaptive pulsatility algorithms designed to reproduce physiological endothelial stimulation while maintaining the durability of continuous-flow devices. Simultaneously, molecular targets involved in mechanotransduction may represent novel therapeutic opportunities to mitigate complications associated with long-term mechanical support.

#### 4.5.4. Pulsatility as a Therapeutic Target

Historically, circulatory support technologies have been designed primarily to restore adequate blood flow. However, accumulating evidence suggests that restoration of flow alone may be insufficient to reproduce the complex biological functions of the native cardiovascular system. The observations summarized in this review support an alternative paradigm in which pulsatility itself constitutes a therapeutic target. Future generations of mechanical circulatory support devices may therefore be evaluated not only according to hemodynamic performance and durability but also according to their capacity to preserve physiological mechanotransduction. Development of adaptive pulsatility algorithms, bioinspired pump designs, and pharmacological strategies targeting mechanosensitive signaling pathways may ultimately bridge the gap between mechanical support and biological compatibility.

The recognition of pulsatility as a fundamental regulator of vascular biology has important translational implications. Rather than focusing exclusively on restoration of systemic blood flow, future circulatory support strategies may seek to preserve or reproduce pulsatility-dependent signaling pathways that maintain endothelial homeostasis and organ function. Advances in mechanobiology, omics technologies, and bioengineered circulatory support systems have identified several potential therapeutic targets that could mitigate the adverse consequences of chronic pulsatility deprivation. The most promising molecular and technological approaches are summarized in Table 6.

The cardiovascular effects of pulsatility operate across multiple biological scales, suggesting that pulsatile flow functions as a systems-level regulator of cardiovascular homeostasis.

The biological consequences of altered pulsatility are unlikely to occur simultaneously. Instead, adaptation to continuous-flow circulation and subsequent restoration of pulsatile hemodynamics following transplantation appear to involve a temporally organized sequence of events. Early responses are predominantly mechanosensory and endothelial, whereas later adaptations involve systemic remodeling and organ-specific consequences. Understanding the chronology of these processes may facilitate identification of optimal therapeutic windows and biomarker-guided interventions. A hypothetical temporal model of pulsatility-dependent biological adaptation is presented in Table 7.

**Figure 7:**
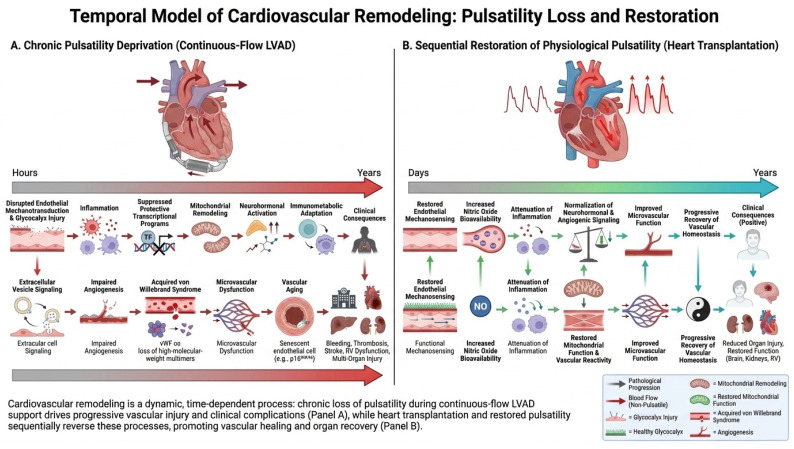
Temporal Model of Cardiovascular Adaptation to Pulsatility Deprivation During Continuous-Flow LVAD Support and Pulsatility Restoration Following Heart Transplantation. Arrow explanations: Horizontal arrows indicate the temporal progression of biological events following chronic loss of pulsatility during continuous-flow LVAD support (Panel (**A**)) and their sequential restoration after heart transplantation (Panel (**B**)). Solid black arrows indicate cause-and-effect relationships or downstream mechanistic pathways. Green arrows indicate recovery or restoration of physiological biological processes. Red arrows indicate pathological progression or adverse biological responses. Brown arrows adjacent to the heart illustrate continuous non-pulsatile blood flow during LVAD support, whereas red pulsatile arrows represent restoration of physiological pulsatile flow after heart transplantation. Upward arrows (↑) denote increased activity, expression, or biological function. Downward arrows (↓) denote decreased activity, expression, or impaired physiological function. Abbreviations: LVAD, left ventricular assist device; NO, nitric oxide; p16^INK4a^, cyclin-dependent kinase inhibitor 2A (senescence marker); TF, transcription factor; vWF, von Willebrand factor. Created with www.figurelabs.ai by Urbanowicz, T. (2026, ID: FL-PUB-20260718-ICW5WS).

Although the molecular and cellular consequences of altered pulsatility have traditionally been described as isolated biological phenomena, accumulating evidence suggests that these responses occur in a temporally organized manner. Adaptation to continuous-flow circulation appears to progress from early mechanosensory and endothelial alterations to more complex vascular, organ-specific, and clinical manifestations over time. Conversely, restoration of physiological pulsatility following heart transplantation initiates a gradual process of biological recovery that may reverse some, but not all, components of cardiovascular remodeling. Understanding the chronology of these adaptations may provide important insights into disease mechanisms, biomarker development, and the identification of optimal therapeutic windows. A proposed temporal framework of pulsatility-dependent cardiovascular adaptation is presented in Figure 7.

#### 4.5.5. Confounding Biological Determinants Beyond Pulsatility

Although pulsatility represents the central mechanobiological framework proposed in this review, many biological alterations observed during continuous-flow LVAD support cannot be attributed exclusively to reduced pulsatile flow. Advanced heart failure itself is associated with chronic neurohormonal activation, systemic inflammation, endothelial dysfunction, oxidative stress, metabolic derangements, cardiac cachexia, renal dysfunction, and hepatic congestion, all of which independently influence vascular biology. Furthermore, device-related factors including supraphysiological shear stress, blood–biomaterial interactions, anticoagulation, infection, and pump-specific characteristics may substantially modify biological responses. Consequently, the vascular phenotype observed during long-term mechanical circulatory support should be interpreted as the result of interacting hemodynamic, inflammatory, metabolic, immunological, and device-related mechanisms rather than pulsatility deprivation alone. Future studies combining carefully matched comparator groups, longitudinal sampling, and multimodal molecular profiling will be required to define the specific contribution of pulsatile hemodynamics to cardiovascular remodeling.

Future translational studies should adopt standardized quantitative measures of pulsatility to enable meaningful comparisons across different LVAD platforms and patient populations. Longitudinal molecular profiling performed before implantation, throughout mechanical support, and after transplantation should be integrated with imaging, hemodynamic parameters, and clinical outcomes to establish causative relationships rather than cross-sectional associations. Harmonized multicenter registries incorporating multi-omics datasets, digital phenotyping, and advanced computational modeling will likely provide the foundation for precision mechanobiology and next-generation circulatory support strategies. Definitive demonstration of causality will require prospective studies combining standardized pulsatility measurements, computational hemodynamics, serial endothelial phenotyping, multi-omics analyses, and comparison of devices generating different pulsatility profiles while controlling for heart failure severity and inflammatory burden.

## 5. Conclusions

The comparison between continuous-flow LVAD support and heart transplantation provides a unique human model for investigating the biological significance of pulsatile circulation. Accumulating evidence suggests that pulsatility regulates endothelial integrity, coagulation homeostasis, inflammatory signaling, microvascular function, and organ adaptation through interconnected molecular pathways. Continuous-flow support induces a distinct vascular phenotype characterized by mechanobiological dysregulation, whereas heart transplantation facilitates partial restoration of physiological signaling networks.

The available evidence suggests that pulsatility may function as an evolutionarily conserved biological signal contributing to endothelial homeostasis, vascular adaptation, and organ function. Continuous-flow LVAD support and heart transplantation therefore provide complementary human models of pulsatility deprivation and restoration. Future integration of mechanobiology, systems biology, and precision medicine approaches may enable development of next-generation circulatory support technologies capable of preserving both mechanical performance and biological compatibility.

## Figures and Tables

**Table 1:** Comparative Biological Consequences of Pulsatility Deprivation During Continuous-Flow LVAD Support and Pulsatility Restoration Following Heart Transplantation (references related to proposed molecular mechanisms).

Biological Domain	Continuous-Flow LVAD (Reduced Pulsatility)	Heart Transplantation (Physiological Pulsatility)	Proposed Molecular Mechanisms	References
Hemodynamics	Continuous or low-pulsatile flow	Physiological pulsatile flow	Restoration of cyclic pressure and shear stress	[23]
Endothelial Function	Endothelial dysfunction	Endothelial recovery	eNOS, KLF2, KLF4 activation	[24,25]
Nitric Oxide Signaling	Reduced NO bioavailability	Increased NO production	Mechanosensitive eNOS regulation	[26,27,28]
Glycocalyx Integrity	Glycocalyx degradation	Potential glycocalyx restoration	Syndecan-1, heparan sulfate signaling	[29,30]
Mechanotransduction	Abnormal signaling	Physiological signaling	Piezo1, YAP/TAZ, PECAM-1 pathways	[31,32]
Oxidative Stress	Increased ROS generation	Reduced oxidative burden	NADPH oxidase and mitochondrial regulation	[33,34]
Inflammation	Persistent inflammatory activation	Partial inflammatory resolution	NF-κB, IL-6, TNF-α	[35]
Hemostasis	Acquired von Willebrand syndrome	Normalization of vWF multimers	Shear-induced vWF cleavage	[36,37]
Angiogenesis	Excessive angiogenic activation	Physiological vascular remodeling	VEGF, Ang-2, HIF-1α	[38,39]
Neurohormonal Activation	Persistent RAAS and SNS activation	Reduced neurohormonal stimulation	Baroreceptor recovery	[40,41,42]
Microcirculation	Impaired autoregulation and capillary recruitment	Improved microvascular function	Endothelial–microvascular coupling	[43,44]
Mitochondrial Function	Bioenergetic dysfunction	Restoration of mitochondrial homeostasis	Oxidative phosphorylation pathways	[45],
Extracellular Vesicles	Increased pro-inflammatory EV release	Reduced vascular activation	Intercellular signaling regulation	[46]
Immunometabolism	Persistent pro-inflammatory phenotype	Partial normalization	mTOR, AMPK, HIF-1α pathways	[47]
Organ Remodeling	Brain, kidney, liver, gut vulnerability	Improved organ perfusion and recovery, gut microbiota changes	Restoration of pulsatile energy transmission	[1,4,48]
Clinical Phenotype	Bleeding, thrombosis, stroke, RV dysfunction	Rejection, vasculopathy, immunosuppression-related complications	Distinct biological adaptations	[49,50]

Abbreviations: LVAD, left ventricular assist device; eNOS, endothelial nitric oxide synthase; KLF2, Krüppel-like factor 2; KLF4, Krüppel-like factor 4; NO, nitric oxide; PECAM-1, platelet endothelial cell adhesion molecule-1; YAP, yes-associated protein; TAZ, transcriptional co-activator with PDZ-binding motif; ROS, reactive oxygen species; NF-κB, nuclear factor kappa B; IL-6, interleukin-6; TNF-α, tumor necrosis factor-alpha; vWF, von Willebrand factor; VEGF, vascular endothelial growth factor; Ang-2, angiopoietin-2; HIF-1α, hypoxia-inducible factor-1 alpha; RAAS, renin–angiotensin–aldosterone system; SNS, sympathetic nervous system; EV, extracellular vesicles; mTOR, mechanistic target of rapamycin; AMPK, AMP-activated protein kinase.

**Table 2:** Biological Determinants of Cardiovascular Remodeling During Continuous-Flow LVAD Support.

Biological Determinant	Principal Mechanism	Representative Consequences	Relative Role	References
Reduced physiological pulsatility	Altered mechanotransduction, reduced oscillatory shear stress	Endothelial dysfunction, impaired NO signaling, microvascular remodeling	Central concept of this review	[61,62]
Advanced heart failure	Neurohormonal activation, chronic hypoperfusion	Oxidative stress, endothelial activation, metabolic dysfunction	Major confounder	[63,64]
Pump-related shear stress	High intrapump shear	vWF degradation, platelet activation, acquired von Willebrand syndrome	Major contributor	[65,66,67]
Systemic inflammation	Cytokine activation, innate immunity	Endothelial injury, vascular remodeling	Major contributor	[68,69]
Blood–biomaterial interaction	Contact activation, complement activation	Thrombosis, inflammatory responses	Device-specific contributor	[70,71]
Comorbidities	Diabetes, CKD, hypertension, aging	Variable endothelial dysfunction and impaired repair	Patient-specific modifier	[72,73]
Genetic and immunometabolic variability	Differences in endothelial reserve and inflammatory responsiveness	Heterogeneous clinical adaptation	Emerging determinant	[74,75]
Device characteristics	Pump design, speed, residual pulsatility	Variable biological responses	Technical modifier	[76,77]

Abbreviations: CKD, chronic kidney disease; NO, nitric oxide; vWF, von Willebrand factor.

**Table 3:** Proposed Hierarchical Model of Pulsatility-Dependent Cardiovascular Regulation.

Biological Level	Continuous-Flow LVAD (Pulsatility Deprivation)	Heart Transplantation (Pulsatility Restoration)	Principal Biological Consequence	References
Mechanosensory Level	Glycocalyx disruption, altered Piezo1 activation	Recovery of physiological mechanosensing	Modified force detection	[169,170]
Molecular Level	Reduced KLF2/eNOS signaling, increased NF-κB activation	Restoration of endothelial homeostasis pathways	Altered gene expression	[171,172]
Organelle Level	Mitochondrial dysfunction, oxidative stress	Mitochondrial recovery	Bioenergetic remodeling	[173,174]
Cellular Level	Endothelial dysfunction, senescence, inflammatory activation	Improved endothelial phenotype	Cellular adaptation	[175,176]
Tissue Level	Vascular remodeling, angiogenesis, fibrosis	Structural normalization	Tissue remodeling	[177,178]
Microcirculatory Level	Impaired autoregulation and capillary recruitment	Improved perfusion matching	Microvascular recovery	[179,180]
Organ Level	Brain, kidney, liver and gut vulnerability	Restoration of organ homeostasis	Organ adaptation	[181,182]
Systemic Level	Persistent neurohormonal and inflammatory activation	Improved physiological integration	Systemic remodeling	[183,184]
Clinical Level	Bleeding, thrombosis, stroke, RV dysfunction	Long-term graft adaptation	Clinical phenotype	[185,186,187]

Abbreviations: LVAD, left ventricular assist device; Piezo1, piezo-type mechanosensitive ion channel component 1; KLF2, Krüppel-like factor 2; eNOS, endothelial nitric oxide synthase; NF-κB, nuclear factor kappa B; RV, right ventricular.

**Table 4:** Evidence Levels for Pulsatility-Dependent Molecular Pathways.

Pathway	Evidence Level	References
vWF signaling and release	Strong	[188,189]
Endothelial dysfunction	Strong	[190,191,192]
Endothelial glycocalyx	Moderate	[193,194]
Piezo1 signaling	Emerging	[195,196]
YAP/TAZ mechanotransduction	Emerging	[197,198]
Immunometabolism	Emerging	[199]
Multi-omics approaches	Hypothesis-generating	[200]

Abbreviations: vWF, von Willebrand factor; Piezo1, piezo-type mechanosensitive ion channel component 1; YAP, yes-associated protein; TAZ, transcriptional co-activator with PDZ-binding motif.

**Table 5:** Strength of Evidence Supporting Pulsatility-Dependent Biological Pathways During Continuous-Flow LVAD Support and Following Heart Transplantation.

Biological Pathway	Human LVAD Evidence	Human HTX Evidence	Experimental Evidence (Animal/In Vitro)	Overall Evidence Strength	Key Knowledge Gaps
Acquired von Willebrand Syndrome	Extensive clinical evidence demonstrating loss of high-molecular-weight vWF multimers and bleeding risk	Rapid normalization following transplantation reported	Strong mechanistic studies of shear-induced vWF cleavage	Strong	Relationship between vWF restoration and vascular remodeling
Endothelial Dysfunction	Reduced NO bioavailability, impaired endothelial function, inflammatory activation reported in multiple studies	Partial recovery of endothelial function and flow-mediated dilation after transplantation	Extensive endothelial mechanobiology literature	Strong	Degree of reversibility after prolonged LVAD support
Angiogenic Dysregulation	Strong association with gastrointestinal angiodysplasia and bleeding	Limited direct evidence of reversal	VEGF, Ang-2, and HIF-1α pathways well characterized experimentally	Strong–Moderate	Mechanisms linking pulsatility to angiogenic signaling
Oxidative Stress and ROS Generation	Increased oxidative stress biomarkers reported in LVAD recipients	Partial reduction after transplantation	Extensive mechanistic evidence	Moderate	Relative contribution of pulsatility versus heart failure biology
Microvascular Dysfunction	Cerebral, renal, and systemic microvascular abnormalities reported	Evidence of partial microvascular recovery	Strong experimental support	Moderate	Lack of longitudinal mechanistic studies
Endothelial Glycocalyx Remodeling	Limited biomarker studies (syndecan-1, glycocalyx shedding)	Virtually no direct evidence	Strong experimental evidence linking flow to glycocalyx integrity	Moderate–Emerging	Direct visualization and longitudinal assessment in humans
Mitochondrial Dysfunction	Indirect evidence from oxidative stress and metabolic studies	Suggested recovery after transplantation	Strong mechanobiology and vascular aging literature	Moderate–Emerging	Human data specifically linking pulsatility to mitochondrial remodeling
Piezo1 Mechanotransduction	No direct clinical LVAD studies	No direct transplantation studies	Strong endothelial mechanosensing evidence	Emerging	Clinical validation in LVAD and transplant populations
YAP/TAZ Signaling	No direct clinical studies	No direct clinical studies	Experimental evidence linking shear stress and endothelial phenotype	Emerging	Human mechanistic studies
Extracellular Vesicles and microRNAs	Several observational biomarker studies	Limited evidence	Increasing experimental support	Emerging	Standardization and validation as biomarkers
Immunometabolic Remodeling	Mostly inferred from inflammatory profiles	Limited indirect evidence	Rapidly expanding experimental literature	Emerging	Direct human mechanistic investigations
Multi-Omics Molecular Signatures	Early transcriptomic/proteomic studies available	Sparse comparative studies	Conceptually supported by systems biology approaches	Hypothesis-Generating	Prospective multi-omics cohorts and validation studies
Reversal of Vascular Aging	Theoretical framework only	Limited indirect evidence from arterial stiffness studies	Experimental support from vascular aging literature	Hypothesis-Generating	Direct demonstration in transplant recipients

Abbreviations: ADAMTS13, a disintegrin and metalloproteinase with thrombospondin motifs 13; ADMA, asymmetric dimethylarginine; AMPK, AMP-activated protein kinase; eNOS, endothelial nitric oxide synthase; EVs, extracellular vesicles; GDF-15, growth differentiation factor 15; HIF-1α, hypoxia-inducible factor-1 alpha; hsCRP, high-sensitivity C-reactive protein; IL-1β, interleukin-1 beta; IL-6, interleukin-6; KLF2, Krüppel-like factor 2; KLF4, Krüppel-like factor 4; LVAD, left ventricular assist device; miR-126, microRNA-126; mTOR, mechanistic target of rapamycin; mtDNA, mitochondrial DNA; NF-κB, nuclear factor kappa B; NO, nitric oxide; NOX2, NADPH oxidase 2; NOX4, NADPH oxidase 4; Nrf2, nuclear factor erythroid 2-related factor 2; oxLDL, oxidized low-density lipoprotein; PECAM-1, platelet endothelial cell adhesion molecule-1; PGC-1α, peroxisome proliferator-activated receptor gamma coactivator 1-alpha; ROS, reactive oxygen species; sGC, soluble guanylate cyclase; SIRT3, sirtuin 3; TNF-α, tumor necrosis factor-alpha; VE-cadherin, vascular endothelial cadherin; VEGF, vascular endothelial growth factor; vWF, von Willebrand factor.

**Table 6:** Pulsatility-Dependent Molecular Targets and Future Therapeutic Opportunities in Mechanical Circulatory Support.

Target Domain	Key Molecular Mediators	Potential Biomarkers	Pathophysiological Consequence of Reduced Pulsatility	Potential Therapeutic Strategy	Translational Readiness	References
Endothelial Mechanotransduction	Piezo1, PECAM-1, VE-cadherin	Endothelial microparticles, soluble PECAM-1	Impaired biomechanical sensing	Piezo1 modulation, mechanosensitive agonists	Experimental	[213,214]
Endothelial Homeostasis	KLF2, KLF4, eNOS	ADMA, NO metabolites	Reduced nitric oxide signaling	eNOS enhancers, NO donors, sGC stimulators	Clinical/Emerging	[215,216]
Glycocalyx Integrity	Syndecan-1, glypicans, heparan sulfate	Syndecan-1, heparan sulfate fragments	Glycocalyx degradation	Glycocalyx-protective therapies	Experimental	[217,218]
Oxidative Stress	NOX2, NOX4, Nrf2	8-isoprostane, oxLDL	Excess ROS generation	Nrf2 activators, mitochondrial antioxidants	Emerging	[219]
Mitochondrial Function	PGC-1α, SIRT3, AMPK	Circulating mtDNA, GDF-15	Bioenergetic dysfunction	Mitochondrial-targeted therapies	Experimental	[220]
Angiogenesis	VEGF, Angiopoietin-2, HIF-1α	VEGF-A, Angiopoietin-2	Angiodysplasia and pathological neovascularization	Anti-angiogenic modulation	Clinical/Emerging	[221]
Hemostasis	vWF, ADAMTS13	vWF multimers, ADAMTS13 activity	Acquired von Willebrand syndrome	Shear-reducing pump design, vWF preservation	Clinical	[222]
Inflammation	NF-κB, IL-6, TNF-α	hsCRP, IL-6	Chronic vascular inflammation	Targeted anti-inflammatory therapies	Clinical	[223]
Immunometabolism	mTOR, HIF-1α, AMPK	Lactate, IL-1β	Persistent inflammatory activation	Metabolic reprogramming strategies	Experimental	[224,225]
Extracellular Vesicles	Endothelial and platelet EVs	Endothelial EVs, platelet EVs, miR-126	Intercellular propagation of injury signals	EV-based biomarkers and therapies	Experimental	[226,227]
Artificial Pulsatility	Variable-speed pump algorithms	Pulsatility Index, SHE	Mechanotransduction impairment	Adaptive pulsatility engineering	Clinical/Emerging	[228,229]
Precision Medicine	Multi-omics biomarkers	Multi-omics molecular signature	Inability to predict complications	Personalized LVAD management	Future	----

Abbreviations: ADAMTS13, a disintegrin and metalloproteinase with thrombospondin type 1 motif 13; ADMA, asymmetric dimethylarginine; AMPK, AMP-activated protein kinase; eNOS, endothelial nitric oxide synthase; EVs, extracellular vesicles; GDF-15, growth differentiation factor-15; HIF-1α, hypoxia-inducible factor-1 alpha; hsCRP, high-sensitivity C-reactive protein; IL, interleukin; KLF2, Kruppel-like factor 2; KLF4, Kruppel-like factor 4; miR-126, microRNA-126; mTOR, mechanistic target of rapamycin; mtDNA, mitochondrial DNA; NF-κB, nuclear factor kappa B; NO, nitric oxide; NOX2, NADPH oxidase 2; NOX4, NADPH oxidase 4; Nrf2, nuclear factor erythroid 2-related factor 2; oxLDL, oxidized low-density lipoprotein; PECAM-1, platelet endothelial cell adhesion molecule-1; PGC-1α, peroxisome proliferator-activated receptor gamma coactivator-1 alpha; ROS, reactive oxygen species; sGC, soluble guanylate cyclase; SHE, surrogate hemodynamic energy; SIRT3, sirtuin 3; TNF-α, tumor necrosis factor-alpha; VEGF, vascular endothelial growth factor; VEGF-A, vascular endothelial growth factor A; vWF, von Willebrand factor.

**Table 7:** Proposed Temporal Model of Biological Adaptation to Pulsatility Deprivation and Restoration.

Time Scale	LVAD Support (Loss of Pulsatility)	Heart Transplantation (Restoration of Pulsatility)	Candidate Biomarkers	References
Minutes–Hours	Altered shear sensing, glycocalyx deformation, Piezo1 activation	Immediate restoration of pulsatile shear stress	Syndecan-1, NO metabolites	[230,231]
Days	eNOS suppression, oxidative stress activation, inflammatory signaling	Recovery of endothelial responsiveness	ICAM-1, VCAM-1, endothelin-1	[232,233]
Weeks	Neurohormonal adaptation, mitochondrial remodeling	Improved vascular reactivity	BNP, renin, catecholamines	[234,235]
Months	Angiogenic activation, acquired vWF syndrome, microvascular dysfunction	Partial reversal of endothelial dysfunction	VEGF, Ang-2, vWF multimers	[236,237]
Years	Organ remodeling, vascular aging, clinical complications	Long-term vascular normalization or transplant vasculopathy	Multi-omics signatures	[238]

Abbreviations: Ang-2, angiopoietin-2; BNP, B-type natriuretic peptide; eNOS, endothelial nitric oxide synthase; ICAM-1, intercellular adhesion molecule-1; LVAD, left ventricular assist device; NO, nitric oxide; VEGF, vascular endothelial growth factor; VCAM-1, vascular cell adhesion molecule-1; vWF, von Willebrand factor.

## Data Availability

No new data were created or analyzed in this study. Data sharing is not applicable to this article.

## Abbreviations

The following abbreviations are used in this manuscript:

ADAMTS13	A Disintegrin and Metalloproteinase with Thrombospondin Motifs 13
ADMA	Asymmetric Dimethylarginine
AMPK	AMP-Activated Protein Kinase
Ang-2	Angiopoietin-2
BNP	B-Type Natriuretic Peptide
CF-LVAD	Continuous-Flow Left Ventricular Assist Device
eNOS	Endothelial Nitric Oxide Synthase
EVs	Extracellular Vesicles
GDF-15	Growth Differentiation Factor 15
HIF-1α	Hypoxia-Inducible Factor-1 Alpha
hsCRP	High-Sensitivity C-Reactive Protein
ICAM-1	Intercellular Adhesion Molecule-1
IL-1β	Interleukin-1 Beta
IL-6	Interleukin-6
KLF2	Krüppel-Like Factor 2
KLF4	Krüppel-Like Factor 4
LVAD	Left Ventricular Assist Device
miR-126	MicroRNA-126
mTOR	Mechanistic Target of Rapamycin
mtDNA	Mitochondrial DNA
NF-κB	Nuclear Factor Kappa B
NO	Nitric Oxide
NOX2	NADPH Oxidase 2
NOX4	NADPH Oxidase 4
Nrf2	Nuclear Factor Erythroid 2-Related Factor 2
oxLDL	Oxidized Low-Density Lipoprotein
PECAM-1	Platelet Endothelial Cell Adhesion Molecule-1
PGC-1α	Peroxisome Proliferator-Activated Receptor Gamma Coactivator 1-Alpha
Piezo1	Piezo-Type Mechanosensitive Ion Channel Component 1
RAAS	Renin–Angiotensin–Aldosterone System
ROS	Reactive Oxygen Species
RV	Right Ventricle
sGC	Soluble Guanylate Cyclase
SHE	Surrogate Hemodynamic Energy
SIRT3	Sirtuin 3
SNS	Sympathetic Nervous System
TAZ	Transcriptional Co-Activator with PDZ-Binding Motif
TNF-α	Tumor Necrosis Factor-Alpha
VCAM-1	Vascular Cell Adhesion Molecule-1
VE-cadherin	Vascular Endothelial Cadherin
VEGF	Vascular Endothelial Growth Factor
vWF	von Willebrand Factor
YAP	Yes-Associated Protein
